# Blood lactate, pH, base excess and pCO_2_ as prognostic indicators in caesarean-born kids from goats with pregnancy toxaemia

**DOI:** 10.1186/s13620-019-0149-1

**Published:** 2019-10-14

**Authors:** I. M. Andrade, P. B. A. Simões, L. P. Lamas, N. Carolino, M. S. Lima

**Affiliations:** 1 CIISA - Centro de Investigação Interdisciplinar em Sanidade Animal, Faculdade de Medicina Veterinária, Universidade de Lisboa, Polo Universitário da Ajuda, 1300-477 Lisbon, Portugal; 2 INIAV, EUVG, 2006-291 Vale de Santarém, Portugal

**Keywords:** Pregnancy toxaemia, Dairy goats, Newborn kids, Lactic acidosis, Survival rate

## Abstract

The objective of this study was to identify the prognostic value for survival of blood parameters in the immediate post-caesarean surgery period in kids born from pregnancy toxaemia (PT) goats. This study involved 10 PT goats, in which a caesarean surgery was performed. Twenty-five kids were born after caesarean surgery of which 16 survived. A blood sample was collected from the jugular vein of the 10 goats and from the kids immediately after caesarean surgery (within 15 min). There were differences between the kids that survived and the kids that did not survive concerning the blood levels of pH (7.22 vs 7.00), base excess (− 9 vs − 18 mmol/L), pCO_2_ (46 vs 62 mmHg) and L-lactate (5.6 vs 16 mmol/L). Maternal ketoacidosis due to PT has a negative impact on the survival rate of the offspring. This appears to be associated to a metabolic acidosis of the offspring. However, the only blood parameter in which there was a strong association between the maternal and newborn kids was blood urea nitrogen (*r* = 0.97).

## Introduction

Pregnancy toxaemia (PT) is a disease that occurs in small ruminants during the last month of gestation when there is a large demand of glucose by the developing foetus (es) [1, 2]. Females (ewes and goats) carrying multiple foetuses are most susceptible [1, 2]. The disease is characterized by a marked metabolic acidosis (ketoacidosis) [1, 2]. Studies in sheep showed that maternal ketoacidosis may result in biochemical and acid-base foetal abnormalities associated with changes in fetoplacental unit perfusion [3].

Several studies have shown blood parameters changes in PT goats. For example, it has been reported a marked increase in L-lactate concentration in the blood collected from the umbilical vein [4], and marked alterations in Na^+^, K^+^, Cl^−^, glucose, pH, HCO_3_^−^, BE, pCO_2_ and BUN, in blood collected from the jugular vein [5]. But unfortunately, literature data on blood parameters of newborn kids from PT goats and their association with a lower survival rate is scarce or even inexistent. In contrast with cattle literature, where a relation of calves blood pH and survival rate is reported, i.e. calves with a blood pH less than 7.2 are considered acidotic, have reduced vitality and consequently a reduced survival rate [6], and a blood pH less than 6.7 is reported not compatible with life [6].

The use of a portable clinical analyzer allows the assessment of blood gases, ions, glucose and BUN levels at the point of care. This analyzer has been employed recently with reliable results in veterinary medicine and allows us to make clinical decisions concerning the goats and their offspring in field conditions [5, 7].

The objective of this study was to identify blood parameters in the immediate post-partum period that can be indicators of prognosis for survival of caesarean-born kids born from PT goats.

## Materials and methods

This study was performed on a 1800-head commercial dairy goat farm, located 40 km northeast of Lisbon, Portugal. The goats were of two breeds: Alpine (approximately 800 animals) and Saanen (approximately 800 animals), with some animals being crosses of these two breeds (approximately 200 animals). All goats were housed all year round. There were three kidding seasons per year, beginning in January, April and October. Each kidding season began on the first day of the month and continued for 45 days. In order to have a group of goats kidding in October (breeding season starting in May), the strategy of the farm is to use implants of melatonin. Daily milk production in this herd averaged approximately 3 L/doe. Machine milking was performed twice daily.

The present study began in September 2016, ended in March 2018, spanned five kidding periods and involved a total of 10 goats diagnosed with PT, (seven Alpine, two Saanen-Alpine crossbred and one Saanen) in which a caesarean surgery was performed. All the goats included in this study were non-lactating. Consent was obtained from the owner of the animals prior to their enrolment in the study. All handling and clinical procedures (blood sampling and caesarean surgeries) realized in PT goats and their offspring occurred as foreseen on the on-farm routine protocol for pregnancy toxaemia. They were performed so as to minimize the stress and discomfort experienced by the animals.

Pregnant goats in the last month of gestation were moved to a pen (dry goat pen) and were fed long-steam hay ad libitum and 1 Kg of a concentrate mix per head/day, divided in four equal portions offered at 8 am, 12 pm, 2 pm and 6 pm. Any pregnant goat in the dry goat pen that refused to eat twice in a row when fresh feed (concentrate) was offered was considered to be a PT suspect**,** and her blood beta-hydroxybutyrate (BHB) concentration was determined using a hand-held electronic on-farm test already validated for quantifying the blood β-hydroxybutyrate concentration in dairy goats (Precision Xceed, Abbott, UK) [8]. If this value was found to be 3 mmol/L, or greater, she was considered to have PT and handled accordingly [2]. The criteria to include a pregnancy toxaemia goat on this study, besides the maternal BHB level, were a blood pH below 7.15 and/or clinical signs such as dropping ears, neurologic signs or recumbency, which are indicators of a poor prognosis [7]. A caesarean surgery was performed in these goats. In order to minimize the associated economic losses due to PT, a clinical strategy adopted by the authors was to induce parturition when the maternal blood pH > 7.15 or to perform a caesarean surgery in the affected goats with a blood pH < 7.15, the goal being to increase the number of live offspring [9].

Once diagnosed with PT, does were enrolled in this study and, a physical examination was performed and a blood sample was obtained from the jugular vein, collected in a 1 ml syringe rinsed with heparin and the following parameters were measured on the farm, using a portable analyser (i-Stat, Sensor Devices Incorporated, Waukesha, WI, USA): Ions (Na^+^, K^+^, Cl^−^ and HCO_3_^−^), glucose, pH, base excess (BE), partial pressure of carbon dioxide (pCO_2_), anion gap (AnGap), blood urea nitrogen (BUN) and L-lactate (The Edge, ApexBio, Hsinchu, Taiwan) [10]. The remaining blood sample was transported in a refrigerated container to laboratory facilities, where it was centrifuged to measure total protein (refractometer - Fisher Scientific, USA) and packed cell volume (PCV).

After the caesarean surgery, a blood sample from the kids was collected in a 1 ml syringe rinsed with heparin from the jugular vein within 15 min (maximal) of being removed from the uterus and before the administration of colostrum. The following parameters were determined at the farm: Na^+^, K^+^, Cl^−^, HCO_3_^−^, glucose, pH, BE, pCO2, AnGap, BUN (i-Stat, Sensor Devices Incorporated, Waukesha, WI, USA) and L-lactate (The Edge, ApexBio, Hsinchu, Taiwan). The remaining blood sample was transported in a refrigerated container to laboratory facilities, where it was centrifuged to measure total protein (refractometer - Fisher Scientific, USA) and packed cell volume (PCV). The number of kids, sex and weight were recorded after birth, as well as the number of kids which died up until the 7th day after birth.

Eight kids were excluded from this study because they were considered to be premature (lack of teeth, hair or open eyes) [2], due to incomplete organs development which will not allow kids to survive.

### Statistical analysis

Pearson’s correlation was used to investigate the association between the blood levels of the several parameters measured in the does and the newborn kids [11]. A two-tailed T test for independent samples was used to compare the mean values of the data obtained from the kids deceased and the kids alive both born from the goats after caesarean surgery [11]. A two tailed Fisher’s exact test was used to determine the significance of differences between the severity of acidosis between the kids which died and kids which were alive after a caesarean surgery [11].

## Results

This study involved 10 PT goats and 25 kids born after a caesarean surgery. All the kids were alive when they were removed from the uterus of the goat. Two of these kids survived for only 10 min, another two kids survived 2 hours, one kid survived 12 h, two kids survived 24 h and two survived 48 h.

At 7 days after birth, of the 10 goats, there were 16 kids alive (64%). Two goats had three kids alive, three goats had two kids alive, one goat had three kids alive and one dead, one goat had one kid alive and one dead, one goat had three dead kids and two goats had two dead kids, making a total of nine dead kids.

The blood chemistry results are summarized in the Tables 1 and 2. The results show a strong correlation between the does and the newborn kids concerning the blood levels of BUN (*r* = 0.97, *p* < 0.001) and a slight association between Na^+^ (*r* = 0.40, *p* < 0.05) and AnGap (*r* = 0.48, *p* < 0.05) (Table 1). There were differences between the 16 survivors and the nine kids that did not survive after a caesarean surgery concerning the blood levels of pH (7.22 vs 7.00, *p* < 0.001), BE (− 9 vs − 18 mmol/L, *p* < 0.05), pCO_2_ (46 vs 62 mmHg, *p* < 0.005) and L-lactate (5.6 vs 16 mmol/L, *p* < 0.001). No statistically significant differences were observed for Na^+^, K^+^, Cl^−^, HCO_3_^−^, total carbon dioxide (tCO_2_), glucose, BUN, AnGap, total protein and PCV (Table 2). It was not possible to obtain the pH blood data from one kid, presumably because the pH was out of the reference range of the analyser (pH < 6.5).

**Table 1:** Blood chemistry values from pregnancy toxaemia goats (*n* = 10) and their kids delivered by caesarean surgery (*n* = 25)

Parameter	Goats Median (range)	Kids Median (range)
**Na** ^**+**^ **(mmol /L)**	134 (123–142)* ***p*** **< 0.05**	138 (121–143) *
K^+^ (mmol /L)	2.8 (2–3.4)	4.2 (3.3–4.8)
Cl^- (^mmol /L)	108 (104–115)	104 (93–112)
Glucose (mmol / L)	2.0 (1.1–3.0)	1.8 (1.1–8.3)
pH	7.12 (6.93–7.29)	7.16 (6.89–7.34) (*n* = 24)
HCO_3_^−^ (mmol /L)	7 (4–13)	19 (12–26) (*n* = 24)
tCO_2_ (mmol /L)	8 (5–14)	20 (13–28) (*n* = 24)
BE (mmol /L)	−23 (− 14 - -28)	−10 (− 20 – − 2) (*n* = 24)
**AnGap (mmol /L)**	20 (10–27)* **p < 0.05**	18 (9–26) * **(*n*** **= 24)**
pCO_2_ (mm /Hg)	23 (16–30)	49 (37–80) (*n* = 24)
PCV (%)	29 (20–50)	48 (29–75)
Total Protein g /dL)	7.9 (7–10)	5 (4.5–5.5)
**BUN (mmol / L)**	5.4 (4.0–18.7)** ***P*** **< 0.001**	6.8 (5.0–17.6) **
BHB (mmol /L)	5 (4.2–8)	NA
L – lactate (mmol /L)	1 (0.3–4.5)	6.5 (3–22)

*AnGap* Anion gap, *BE* Base excess, *BUN* Blood urea nitrogen, *NA* Not applicable, *pCO2* partial pressure of carbon dioxide, *PCV* Packed cell volume, *PT* Pregnancy toxaemia, *tCO2* total carbon dioxide. Significance level at *P < 0.05, **P < 0.001

The parameters (Na^+^, AnGap and BUN) in which there is an association between the maternal and foetal blood levels are represented in bold

**Table 2:** Blood chemistry values from kids that survived (SK; *n* = 16) and died (DK; *n* = 9) before 7 days of age

Parameter	SK kids Median (range)	DK kids Median (range)
Na^+^ (mmol /L)	138 (128–142)	138 (121–143)
K^+^ (mmol /L)	4.2 (3.3–4.8)	4.4 (3.8–4.8)
Cl^−^ (mmol /L)	104 (96–112)	104 (93–111)
Glucose (mmol /L)	1.4 (1.1–7.5)	2.8 (1.1–8.3)
pH	7.22 (6.89–7.34)	7.00 (6.89–7.15)*** (*n* = 8)
HCO_3_^−^ (mmol /L)	19 (15–25)	19 (15–25) (*n* = 8)
tCO_2_ (mmol /L)	21 (16–26)	15 (12–26) (*n* = 8)
BE (mmol /L)	−9 (− 18 - -2)	−18 (− 21 – - 3)* (*n* = 8)
AnGap (mmol /L)	18 (12–23)	20 (9–26) (*n* = 8)
pCO_2_ (mmHg)	46 (37–80)	62 (48–75)** (*n* = 8)
PCV (%)	48 (34–75)	52 (29–55)
Total Protein (g /dL)	5 (4.6–5.5)	5 (4.5–5)
BUN (mmol/ L)	7.2 (3.6–8.6)	6.5 (5.0–17.6)
L – lactate(mmol /L)	5.6 (2.8–10)	16 (6.3–22)***

*AnGap* Anion gap, *BE* Base excess, *BUN* Blood urea nitrogen, *pCO2* partial pressure of carbon dioxide, *PCV* Packed cell volume, *SK* Kids that survived, *DK* Kids that died, *tCO2* total carbon dioxide. Significance level at *P* < 0.05, *P < 0.05, **P < 0.005, ***P < 0.001

## Discussion

Perinatal mortality is a problem in sheep and goats worldwide and may have regional and seasonal variations, depending on the relation between environment, pathogens and the host [12–15]. A recent study in dairy goats raised in an intensive system showed that PT is one of the most common diseases contributing to neonatal mortality with a negative impact on the survival rate of the offspring [6]. The authors reported a seven-day survival percentage of 77% (20 out of 26) in kids delivered by caesarean surgery from PT goats [6].

Haematological and biochemical parameters in the immediate post-partum period have been demonstrated to have significant prognosis value in calves [4] and lambs [3]. The results of our study show that in newborn kids, decreased blood pH, decreased blood BE, increased blood L-lactate and increased blood pCO_2_ are associated with a poor prognosis. According to the blood pH (7.00 vs 7.22), base excess (− 18 vs − 9 mmol/L), and L-lactate values (16 vs 5.6 mmol/L), the metabolic (lactic) acidosis was more severe in non-surviving kids. These kids also showed a moderate increase in pCO_2_ (62 vs 46 mmHg). These findings are in line with the humans [16], and cattle [17] literature that showed that a metabolic acidosis at birth can affect the vitality and the rate of survival of the newborn. All the kids which died within 7 days of birth were acidotic (pH < 7.15, 9/9, 100%), and 14 kids that survived were also acidotic (pH < 7.30, 14/16, 88%). Even so, blood pH values in kids that died were significntly lower than in kids that survived (*p* < 0.001), indicating more severe acidosis. The lowest pH in the kids that survived from pregnancy toxaemia goats was 6.89.

Increased blood levels of L-lactate were also reported in neonatal calves in which there was a strong correlation between blood pH and L-lactate [4]. In humans, L-lactate and acid base balance have similar predictive value concerning adverse foetal outcome but the simplicity and low cost makes L-lactate an interesting alternative to conventional acid-base analysis in obstetric care [18]. This information is important because L-lactate analyser can be easily managed by clinicians at farms and is considerably less expensive to measure blood L-lactate than blood pH (with the i-Stat). In commercial dairy goat farms, we recommend to evaluate the blood levels of L-lactate in kids born from PT goats after a caesarean surgery that are weak, lethargic, that do not vocalize and with a weak or absent suction reflex. These kids are likely to be acidotic, therefore have a guarded prognosis and should be treated appropriately.

In this study, blood parameters measured are characteristic of goats with PT which have been thoroughly reported in a previous study [5]. There was a strong association between the maternal and newborn kids concerning blood BUN (*r* = 0.97). This result may be explained due to the crossing of urea through the placental barrier by diffusion, the high permeability of the ovine placenta increasing from the 80th day of gestation to term at a rate roughly proportional to that of foetal weight [19]. Our results also demonstrate a slight association between maternal and newborn blood levels of Na^+^ (*r* = 0.40) and AnGap (*r* = 0.48), however these associations do not appear to contribute to the survival prognosis of the kids. Finally, no association was seen between the blood pH, BE, pCO_2_ and L-lactate of the doe and the newborn kids.

Although maternal ketonaemia of multiple-foetus pregnancies is accompanied by increased uterine uptake of BHB, little or no BHB is transferred to the foetus, indicated that ketone bodies become an important energy source for the placenta but not for the foetus. The type of placentation in the goats (syndesmochorial placentation) prevents the transfer of maternally-derived BHB to the foetal circulation and therefore its impact on foetal acid-base balance is minimal [16, 20].

## Conclusions

Although this study was based on a small sample size (10 goats and 25 newborn kids), the results show that in newborn kids born from PT goats by caesarean surgery, decreased blood pH, decreased blood BE, increased blood L-lactate and increased blood pCO_2_ are associated with a poor prognosis. It would be a laudable to evaluate the impact of the administration of fluids supplemented with sodium bicarbonate on the rate of survival of these newborn kids. Further studies are also needed to compare blood parameters of healthy neonate kids with kids from PT.

## Data Availability

The datasets used and analysed during the current study are available from the corresponding author on reasonable request.

## Abbreviations

AnGap: Anion gap
BE: Base excess
BHB: Beta-hydroxybutyrate
BUN: Blood urea nitrogen
pCO_2_: Partial pressure of carbon dioxide
PCV: Packed cell volume
PT: Pregnancy toxaemia
tCO_2_: total carbon dioxide
